# Cost-effectiveness analysis of surgical masks, N95 masks compared to wearing no mask for the prevention of COVID-19 among health care workers: Evidence from the public health care setting in India

**DOI:** 10.1371/journal.pone.0299309

**Published:** 2024-05-20

**Authors:** Meenakshi Sharma, Harnoor Sra, Chris Painter, Wirichada Pan-ngum, Nantasit Luangasanatip, Anil Chauhan, Shankar Prinja, Meenu Singh

**Affiliations:** 1 Queens University, Belfast, United Kingdom; 2 Post Graduate Institute of Medical Education and Research, Chandigarh, India; 3 Faculty of Tropical Medicine, Mahidol-Oxford Tropical Medicine Research Unit, Mahidol University, Bangkok, Thailand; 4 Health Intervention and Technology Assessment Program (HITAP), Ministry of Public Health, Nonthaburi, Thailand; 5 Nuffield Department of Medicine, Centre for Tropical Medicine and Global Health, University of Oxford, Oxford, United Kingdom; 6 All India Institute of Medical Sciences, Rishikesh, India; North South University, BANGLADESH

## Abstract

**Background:**

Nonpharmacological interventions, such as personal protective equipment for example, surgical masks and respirators, and maintenance of hand hygiene along with COVID-19 vaccines have been recommended to reduce viral transmission in the community and health care settings. There is evidence from the literature that surgical and N95 masks may reduce the initial degree of exposure to the virus. A limited research that has studied the cost-effective analysis of surgical masks and N95 masks among health care workers in the prevention of COVID-19 in India. The objective of this study was to estimate the cost-effectiveness of N95 and surgical mask compared to wearing no mask in public hospital settings for preventing COVID-19 infection among Health care workers (HCWs) from the health care provider’s perspective.

**Methods:**

A deterministic baseline model, without any mask use, based on Eikenberry et al was used to form the foundation for parameter estimation and to estimate transmission rates among HCWs. Information on mask efficacy, including the overall filtering efficiency of a mask and clinical efficiency, in terms of either inward efficiency(ei) or outward efficiency(e0), was obtained from published literature. Hospitalized HCWs were assumed to be in one of the disease states i.e., mild, moderate, severe, or critical. A total of 10,000 HCWs was considered as representative of the size of a tertiary care institution HCW population. The utility values for the mild, moderate and severe model health states were sourced from the primary data collection on quality-of-life of HCWs COVID-19 survivors. The utility scores for mild, moderate, and severe COVID-19 conditions were 0.88, 0.738 and 0.58, respectively. The cost of treatment for mild sickness (6,500 INR per day), moderate sickness (10,000 INR per day), severe (require ICU facility without ventilation, 15,000 INR per day), and critical (require ICU facility with ventilation per day, 18,000 INR) per day as per government and private COVID-19 treatment costs and capping were considered. One way sensitivity analyses were performed to identify the model inputs which had the largest impact on model results.

**Results:**

The use of N95 masks compared to using no mask is cost-saving of $1,454,632 (INR 0.106 billion) per 10,000 HCWs in a year. The use of N95 masks compared to using surgical masks is cost-saving of $63,919 (INR 0.005 billion) per 10,000 HCWs in a year. the use of surgical masks compared to using no mask is cost-saving of $1,390,713 (INR 0.102 billion) per 10,000 HCWs in a year. The uncertainty analysis showed that considering fixed transmission rate (1.7), adoption of mask efficiency as 20%, 50% and 80% reduces the cumulative relative mortality to 41%, 79% and 94% respectively. On considering ei = e0 (99%) for N95 and surgical mask with ei = e0 (90%) the cumulative relative mortality was reduced by 97% and the use of N95 masks compared to using surgical masks is cost-saving of $24,361 (INR 0.002 billion) per 10,000 HCWs in a year.

**Discussion:**

Both considered interventions were dominant compared to no mask based on the model estimates. N95 masks were also dominant compared to surgical masks.

## Introduction

Coronavirus disease 2019 (COVID-19) is a highly transmissible virus and was declared a pandemic by the World Health Organization (WHO) on March 11, 2020 [1]. COVID-19 spreads person-to-person through respiratory droplets, fomites, and contacts [2]. COVID–19 infected individuals can initially be asymptomatic and may later develop mild to severe respiratory symptoms and die [3]. Globally, measures such as lockdowns, social distancing, contact tracing, and enhanced surveillance have been employed to reduce person-to-person transmission [4]. Nonpharmacological interventions (NPIs), such as personal protective equipment (PPE), for example, surgical masks and respirators, and maintenance of hand hygiene along with COVID-19 vaccines have been recommended to reduce viral transmission in the community and health care settings [5]. A simulation study on COVID-19 emphasized that vaccine alone is insufficient to contain the pandemic and the risks associated with relaxation of NPIs [5]. The Centres for Disease Control and Prevention (CDC), United States has also issued guidelines promoting double masking, which emphasized the use of masks for the prevention of COVID-19 infection [6,7]. The WHO advocates surgical masks for non-aerosol generating procedures involving COVID-19 patients but the CDC, United States and European CDC differ and recommend the use of N95 respirators even for non-aerosol generating procedures [8]. N95 and surgical masks are both used by health care workers (HCWs) in India who treat COVID‐19 patients. An Indian study reported that the majority (60.16%) of the HCWs use triple-layered masks as compared to N95 (12.03%) and cited non-availability of N95 masks or strict implementation of rational use policy [9]. The HCWs are highly susceptible to COVID-19 infections and has a high probability of transmitting infections to the general population without personal protective equipments, yet research on the cost effectiveness strategies is limited [10,11].There is also evidence from the literature that surgical and N95 masks may reduce the initial degree of exposure to the virus, and that they were effective in reducing the risk of infection of the severe acute respiratory syndrome (SARS) [12]. The rates of both laboratory confirmed bacterial/viral colonisation and droplet transmitted infections (RR 0.33, RR 0.46, RR 0.26) found significantly lower in the N95 arms [13]. A meta-analysis has shown the protective effect of respirators (OR = 0.12; 95% CI: 0.06–0.26) and masks (OR = 0.13; 95% CI: 0.03–0.62) against SARS [14]. Therefore, the importance of NPIs should not be undermined, especially among HCWs. As a result of disproportionate use of PPE in a resource-constrained country like India, supply and demand may differ substantially, which could result in a shortage of PPE. In addition, there is limited research that has studied the cost-effective analysis of surgical masks and N95 masks among HCWs in the prevention of COVID-19 in India. A cost-effectiveness analysis of hand-hygiene, N-95 respirators, and the surgical masks has been conducted but among the general population, rather than HCWs [11]. The study showed that hand hygiene, the use of surgical mask, respirator, and surgical mask with hand hygiene has significant clinical effectiveness though not cost-effective [11]. As a result, the objective of this study was to estimate the cost-effectiveness of N95 and surgical mask compared to wearing no mask in public hospital settings for preventing COVID-19 infection among HCWs from the health care provider’s perspective.

## Methods

A cost-effectiveness analysis (CEA) was conducted using a SIR (Kermack-McKendrick) type model, driven by deterministic differential equations from Eikenberry et al [15] to mimic disease transmission. Costs and outcomes (quality-adjusted life-years [QALYs] were calculated to compare the cost-effectiveness of N95 masks, surgical masks and no masks with each other for the prevention of COVID-19 infection) among the HCWs. HCWs were chosen as the population for this study because HCWs are among those with the highest risk of infection in the general population [16]. The model considered N95 masks and surgical masks as the intervention options against and no masks. The model had a one-year simulation, one day cycle length and the analysis was performed from a health care provider’s perspective. The modelled time period was one year to restrict the analysis to the emergency phase of the pandemic. Longer term projections were not considered feasible given the uncertainty of the impact of other interventions (such as vaccination) and acquired immunity. No discounting was performed as all costs were assumed to accrue in the first year. The incremental cost-effectiveness ratio (ICER), in Indian Rupees (INR) per QALY gained, was calculated. Based on WHO guidelines for cost-effectiveness thresholds (CETs), a gross domestic product (GDP) per capita-based threshold was applied [17]. India’s GDP per capita (INR 142,719) considered as the cost-effectiveness threshold value [18] and less than one GDP per capita ICER was considered highly cost-effective [11]. The appropriateness of the GDP per capita threshold have also been disputed, and therefore alternative CETs of $115-$770 per QALY were also considered based on estimates from Woods et al. for India [19]. The model was developed using Microsoft Office Excel 2020. The model accuracy was verified by reproducing similar results to the Eikenberry et al. paper when the model inputs supplied in the manuscript were used [15]. This study follows the recommendations of the Consolidated Health Economic Evaluation Reporting Standards (CHEERS) reporting guideline.

### Model

**Fig 1:**
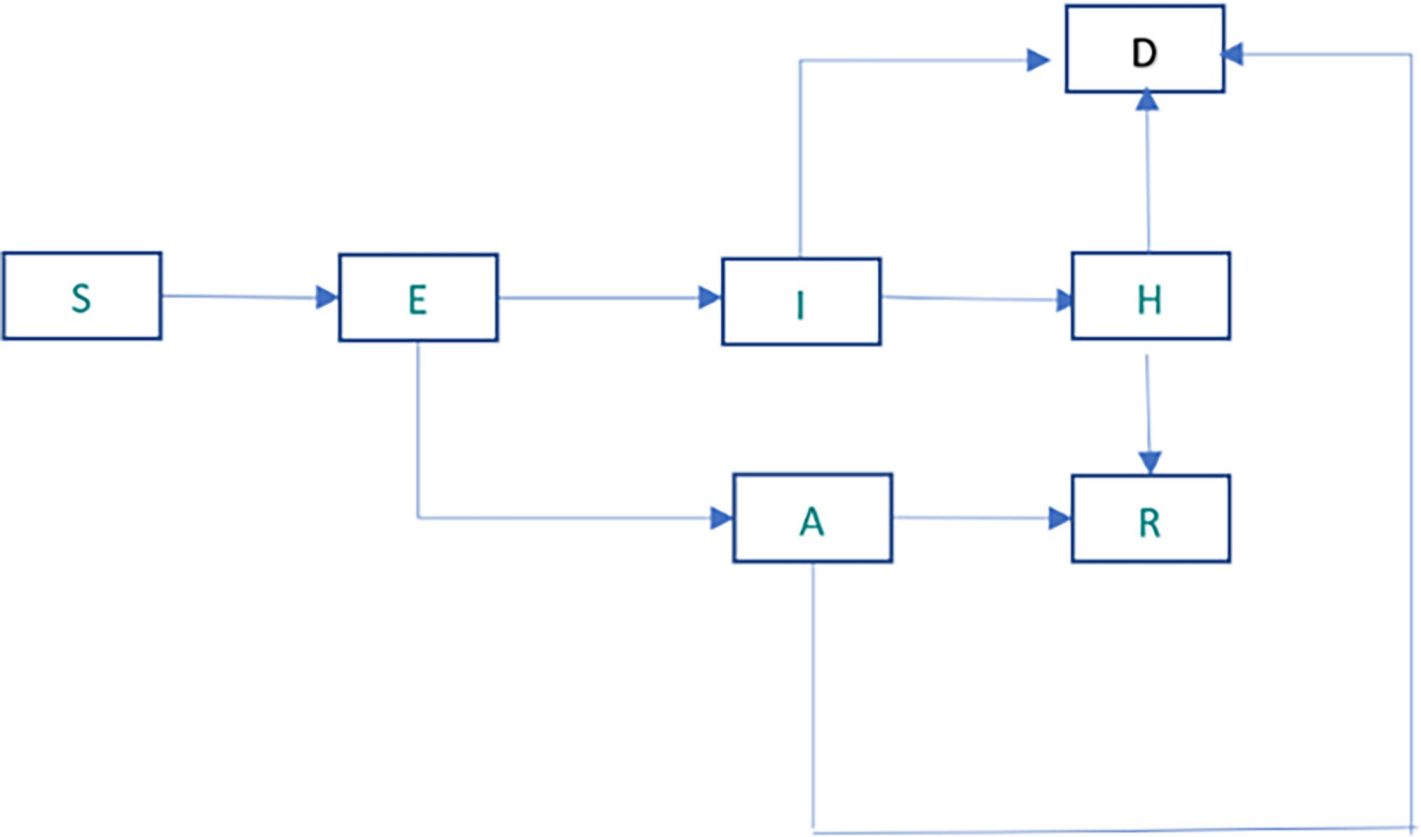
SEIAHRD model based on Eikenberry et al.

A deterministic baseline model, without any mask use, based on Eikenberry et al [15] was used to form the foundation for parameter estimation and to estimate transmission rates among HCWs. In the present study we adapted Eikenberry et al [15] as this is based on previously developed SEIR model frameworks for transmission dynamics. Moreover, the model explores the potential impact of face masks of varying efficacy and compliance on the transmission dynamics and control of the COVID-19. We considered hospital settings as a community in our present study. The model structure included the following compartments at any time t: susceptible S(t), exposed E(t), symptomatic infectious I(t), hospitalized H(t), asymptomatic infectious A(t), recovered R(t) and cumulative deaths D(t) (Figs 1 and S1). It was assumed that some fraction of detected infectious HCWs would progress to the hospitalized compartment H(t), where it was assumed that hospitalized HCWs would not be exposed to the general population and thus do not contribute to the infection risk for susceptible to others. It was also assumed that HCWs abstained from going to work once infected. No interactions and transmission between HCWs and non HCWs were assumed based on the evidence that HCWs during COVID-19 moved out of their homes to ensure physical distance and minimise the risk of transmission to others [20].

Although a certain fraction of hospitalized HCWs may eventually need critical care and die, the model does not explicitly distinguish between ICU and non-ICU patients. Due to the short time horizon, a closed population was assumed in the model, where natural births and all cause deaths were not accounted for.

HCWs were distributed across the susceptible compartments at the model start for both N95, surgical mask were 950(don’t wear mask),9500(wear mask) and in the and symptomatic infectious compartment were 50 respectively. Whereas for no mask arm at the model start, HCWs were distributed as 9,950 and 50 in susceptible and symptomatic infectious compartments.

Our model did not include HCWs involved in the direct treatment of COVID-19 patients, who are already mandated to wear PPE. Furthermore, this model was used to determine the equivalent transmission rate reductions resulting from either N95 or surgical face masks.

The severity of COVID-19 was defined according to WHO classifications:[21]

- **Mild disease**: Symptomatic patients meeting the case definition for COVID-19 without evidence of viral pneumonia or hypoxia
- **Moderate disease—pneumonia:** Adolescent or adult with clinical signs of pneumonia (fever, cough, dyspnoea, fast breathing) but no signs of severe pneumonia, including SpO2 ≥ 90% on room air
- **Severe disease—severe pneumonia:** Adolescent or adult with clinical signs of pneumonia (fever, cough, dyspnoea, fast breathing) plus one of the following: respiratory rate > 30 breaths/min; severe respiratory distress; or SpO2 < 90% on room air
- **Critical disease**: Acute respiratory distress syndrome, Acute thrombosis, sepsis, septic shock, Multisystem Inflammatory Syndrome in Children.

### Model parameters

Table 1 shows the epidemiological parameters used in the model which were taken from published literature [15,22–25]. The assumptions and simplifications for the transmission dynamics of COIVD-19 were based on the adapted model [15]. β is the baseline infectious contact rate. β is assumed to vary with time but usually considered as fixed. For the current study the fixed β was set. The η represents the relative infectiousness of asymptomatic carriers in comparison to symptomatic carriers. The 1/σ is considered as the disease incubation period as σ is the transition rate from the exposed to infectious. The α accounts for the fraction of cases that are symptomatic and φ is the rate at which symptomatic are hospitalized. The γA, γI and γH are recovery rates for the subscripted HCWs and δ is the disease-induced death rate. Information on mask efficacy, including the overall filtering efficiency of a mask and clinical efficiency, in terms of either inward efficiency(ei) or outward efficiency(e0), was obtained from published literature (Table 2). Hierarchy of evidence-based study designs was considered for probability data from published literature. Therefore, probabilities from systematic review and meta-analysis were prioritized, followed by randomized controlled trials and then observational studies. Approximately, 90% HCWs were assumed to wear masks in the N95 and surgical mask intervention arms of the model, based on a cross-sectional study that was conducted during the peak of COVID-19 pandemic to explore the compliance rate to mask-wearing among HCWs in a tertiary care hospital, which found that approximately 90% HCWs were compliant. The compliance may not remain the same after the pandemic is over. Hence, no mask scenario was also kept in the model. All variables were disaggregated for those wearing masks, and those not, illustrated by a subscript M and U, respectively. Hospitalized HCWs were assumed to be in one of the disease states i.e., mild, moderate, severe, or critical. A total of 10,000 HCWs was considered as representative of the size of a tertiary care institution HCW population [26]. The hospitalized compartment (H_U_+H_M_) was distributed into moderate (40%), severe (38%), and critical (22%) health states, based on published literature in the Indian context [27]. Based on Zhou et al, the average length of stay for hospitalized cases was considered to be 12, 7.5, and 8 days for the moderate, severe, and critical health states, respectively [28]. The average age of 40.2 years was considered for calculating life years lost due to death, based on evidence from nurses employed in a tertiary care institution [29]. In a tertiary care hospital, nurses are the largest fraction of the overall population. Hence, average age of the nurses was taken to calculate years of life lost.

**Table 1:** Epidemiological parameters.

	Parameter value	Reference
Transmission rate (β)	1.67 (1.47–1.86)*	(Gupta M et al., 2021)[22]
Transition exposed to infectious (σ)	0.58	(Wassie GT et al., 2020)[23]
Infectiousness factor for asymptomatic carriers(η)	0.4–0.6	(Eikenberry SE et al., 2020)[15]
Fractions of infections that become symptomatic(α)	0.15–0.7	
Rate of hospitalization(ϕ)	0.02	(Srivastav AK 2021 et al., 2021)[25]
Recovery rate (asymptomatic) (γa)	1/7 day^-1^	(Eikenberry et al., 2020)[15]
Recovery rate (symptomatic) (γI)	1/7 day^-1^	
Recovery rate (hospitalized) (γH)	1/14 day^-1^	
The death rate of hospitalized(δ)	0.0042	(Srivastav AK2 et al., 2021)[25]
Reproductive number (R_0_)	1.38 (1.31–1.38)*	(Marimuthu et al., 2021)[24]

* Point estimates added if available in the literature.

**Table 2:** Mask efficiency parameters.

Mask parameter	Parameter value	Reference
*Inward mask efficiency (ei)*		
Surgical mask	70–90%	(Eikenberry et al., 2020)[15]
N95	95%	
*Outward mask efficiency(eo)*		
Surgical mask	50–90%	(Eikenberry et al., 2020)[15]
N95	70–100%	

### Utility data

The utility values for the mild, moderate and severe model health states were sourced from the primary data collection on quality-of-life of HCWs COVID-19 survivors. The study was conducted at a tertiary care hospital as primary research for this economic evaluation. The data was collected using the EQ-5D-5L questionnaire [30]. The authors conducted a cross-sectional survey (June–August 2021) engaging HCWs of a tertiary care hospital in northern India who got infected COVID-19 during this period. A list of HCWs were taken from the hospital records. The consent was obtained by telephone from patients who had recovered from COVID-19 in the period of 2 weeks to avoid recall bias. A link to a questionnaire which requested simple demographic information and the EQ-5D-5L questionnaire was shared via Google to those who gave consent to participate. In an expert group meeting, it was decided to target a sample size of 100. The sample size obtained was mild (n = 69), moderate (n = 27) and severe (n = 20) respectively. It was not possible to get any patients who were deemed critical and who survived. The utility scores for mild, moderate, and severe COVID-19 conditions were 0.88, 0.738 and 0.58, respectively. As no value set is currently available for the Indian population, the value-set for Thailand was used to determine the utility value for the HCW’s health states (mild, moderate and severe) [31]. QALYs lost due to time spent in the COVID-19 infection health states (mild, moderate, severe, and critical) were calculated. The utility score for critical severity was assumed to be the same as severe COVID-19 health state as no patient could be registered in that state during the survey. Total QALYs lost due to mortality were calculated by subtracting the average age of HCWs from the average life expectancy in India of 67 years [32]. The total number of deaths averted in one year were estimated from the total number of COVID-19 deaths reported in the intervention arm in one year minus the total number of deaths in the comparator arm in the same year and this value was totalled with the QALYs averted obtained from respective health states.

### Cost data

The mask costs per health personnel in the risk zones were calculated by assuming that 2 masks (N95/surgical masks) were provided per health personnel for each day (Table 3). The cost of no mask was zero. Table 3 also shows the cost of treatment for mild sickness (6,500 INR per day), moderate sickness (10,000 INR per day), severe (require ICU facility without ventilation, 15,000 INR per day), and critical (require ICU facility with ventilation per day, 18,000 INR) per day as per government and private COVID-19 treatment costs and capping [33]. A conversion rate of 1 INR = 0.014 USD was applied to all costs.

**Table 3:** Cost parameters.

Parameter	Cost USD (INR)	Source
Cost of N95 masks (2 masks per person per day)	1.34 (95.6)	State government medical services corporation for bulk purchasing of drugs of a tertiary care institution
Cost of surgical masks (2 masks per person per day)	0.22 (15.7)	State government medical services corporation for bulk purchasing of drugs of a tertiary care institution
Cost of treatment (per person per day)		
Cost of asymptomatic disease	0	Package rates of Government and private COVID-19 treatment costs and capping in Haryana and Punjab states of India
Cost of mild disease	91 (6500)	
Cost of moderate disease	140 (10,000)	
Cost of severe disease	210 (15,000)	
Cost of critical	252 (18,000)	

### Model validation and one way sensitivity analysis

The model structure and some transmissibility parameters were validated elsewhere against the cumulative mortality data [15]. Other parameters are fixed at default values in Table 1. The population size of 10,000 HCWs was considered as representative of the size of a tertiary care institution HCW population. One way sensitivity analyses were performed to identify the model inputs which had the largest impact on model results, the most impactful individual model inputs were the parameters related to the inward and outward efficiency of N95 and surgical masks. The inward efficiency of N95 and surgical masks were varied in scenario analyses from 0.95 to 0.99, and 0.70 to 0.90, respectively. The outward efficiency of N95 were varied in scenario analyses from 0.70 to 0.99. The outward efficiency of the surgical masks varied from 0.50 to 0.90. A further analysis was also performed assuming the inward(ei) and outward efficiency(e0) to be same (ei = e0 = e) as 20%, 50% and 80% for both N95 and surgical masks respectively and findings were compared with the no mask scenario.

## Results

### Cost-effectiveness analysis

**Fig 2:**
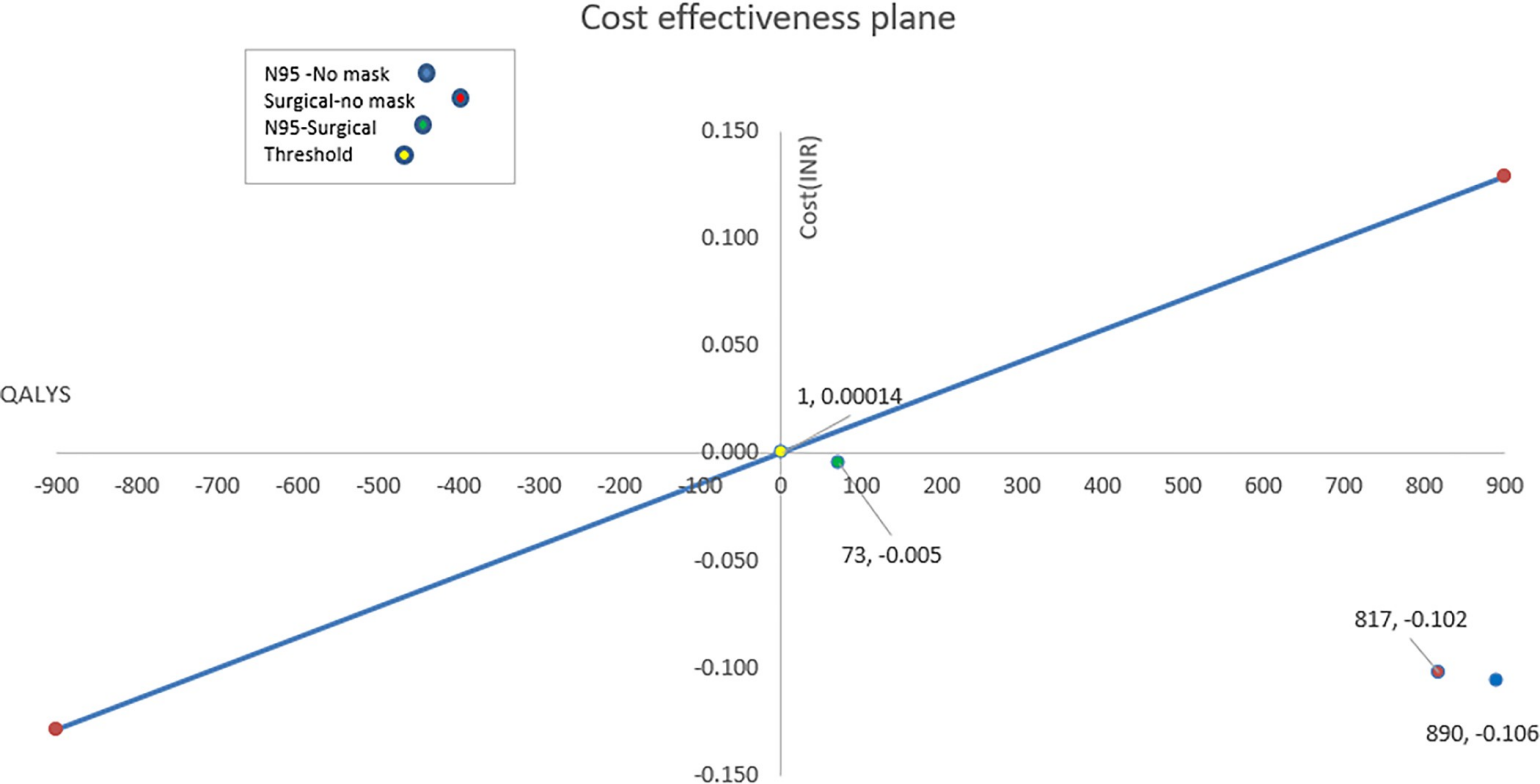
Cost-effectiveness plane with incremental cost-effectiveness ratios.

Fig 2 displays the ICERs for each of the comparisons between the interventions and comparators plotted on a cost-effectiveness plane.

### Surgical mask vs no mask

Based on the health system perspective, the use of surgical masks compared to using no mask is cost-saving of $1,390,713 (INR 0.102 billion) per 10,000 HCWs in a year. The total costs estimated for the surgical mask intervention were $124,274 (INR 0.009 billion) per 10,000 HCWs, compared to $1,514,988 (INR 0.108 billion) in the no mask arm. The annual incidence of deaths in the surgical mask was 3.92 as compared to 34.27 in using no intervention. Additional QALYs gained among incidence cases for the surgical masks in comparison to no mask were 817 with 30 deaths averted.

#### N95 mask vs no mask

The use of N95 masks compared to using no mask is cost-saving of $1,454,632 (INR 0.106 billion) per 10,000 HCWs in a year. The total costs estimated for the N95 mask intervention were $603,55 (INR 0.004 billion) per 10,000 HCWs, compared to $1,514,988 (INR 0.108 billion) in the no mask arm. The incidence of deaths in the N95 mask was 1.20 as compared to 34.27 in using no intervention. Additional QALYs gained among incidence cases for N95 masks in contrast to no mask were 890 with 33 deaths averted. The incidence of deaths in the N95 mask was 1.20 as compared to 34.27 in using no intervention.

#### N95 vs surgical mask

The use of N95 masks compared to using surgical masks is cost-saving of $63,919 (INR 0.005 billion) per 10,000 HCWs in a year. The total costs estimated for the surgical mask intervention were $1,242,74 (INR 0.009 billion) per 10,000 HCWs, compared to $603,56 (INR 0.004 billion) in the N95 arm. The incidence of deaths in the N95 mask was 1.20 as compared to 3.9 in using surgical masks. Additional QALYs gained among incidence cases for N95 masks in comparison to surgical masks were 73 with 3 deaths averted. Therefore, N95 masks were estimated to dominate surgical masks, as N95 masks resulted in reduced costs and superior health outcomes than surgical masks.

The model estimated that both N95 and surgical masks were cost-effective compared to no mask (Fig 2). The ICER fell in the southeast quadrant, where wearing an N95 or surgical mask resulted in better health outcomes and lower costs than no mask. The QALYs gained for the mild COVID-19 health state among incidence cases in the N95, surgical mask compared to no mask was estimated to be 15.78 and 14.50 respectively (S1 Table). In the scenario of no masking among HCWs, starting from a baseline of 9,950 susceptible and 50 infected (symptomatic infectious) HCWs, the incidence cases of hospitalized (617), asymptomatic infectious (4,974) and deaths [34] are shown in Table 4.

**Table 4:** Cost-effectiveness results.

	No mask	N95	Surgical mask
A. Model estimation			
Total cost (USD) (INR in billions)	1,514,988 (0.108)	60,356 (0.004)	1,242,75 (0.008)
Incremental cost*(USD) (INR in billions)	-	1,454,632 (0.106)	1,390,713 (0.102)
Hospitalized cases	617	21	43
Mild cases	5,024	127	253
B. Incident cases			
Hospitalized cases	617	21	43
Mild cases	5,024	127	525
Total cases (mild + asymptomatic)	9,998 (5,024 + 4,974)	254 (127 + 127)	778 (525 + 253)
Cases prevented*	-	9,744	9,220
Death	34	1	4
C. QALYS		890	817

* compared to no mask.

* cost in billions.

## Uncertainty analysis

As the inward and outward efficiency of N95 and surgical masks were the most impactful model parameters on the results, the results of these scenarios have been reported. On considering a fixed transmission rate of β (1.7), N95 mask with ei = e0 (99%), surgical mask with ei = e0 (90%) the cumulative relative mortality was reduced by 97%. On considering a fixed transmission rate of of β (1.7), N95 mask with ei = e0 (99%), surgical mask with ei = e0 (90%) the cumulative relative mortality was reduced by 97%. The use of N95 masks compared to using surgical masks is cost-saving of $24,361 (INR 0.002 billion) per 10,000 HCWs in a year. The use of N95 masks compared to using no mask is cost-saving of $14,664,37(INR 0.107 billion) per 10,000 HCWs in a year. The use of surgical masks compared to using no mask is cost-saving of 14,420,76(INR 0.105 billion) per 10,000 HCWs in a year. Considering a fixed transmission β (1.7) with e as 20%, 50%, and 80% reduced the cumulative relative mortality by 41%, 79% and 94% respectively. On varying the transmission rate (1.9), the use of N95 masks compared to surgical masks was estimated to be cost saving by $83,181 (INR 0.006 billion) per 10,000 HCWs in a year. On decreasing the transmission rate (1.5), the use of N95 masks compared to surgical masks was cost saving by $63,919 (INR 0.005 billion) per 10,000 HCWs in a year. A one-way sensitivity analysis, where key parameters were varied individually to10% higher and lower values from their base case values was conducted. The varied parameters were: transition exposed to infectious, infectiousness factor for asymptomatic carriers(η), fractions of infections that become symptomatic(α), rate of hospitalization(ϕ), rate of hospitalization(ϕ), recovery rate (asymptomatic) (γa), recovery (symptomatic) (γI), recovery rate (hospitalized) (γH) and the death rate of hospitalized(δ). The N95 masks remained cost-effective in all tested scenarios with respect to variations in the above-mentioned key input parameters. The utility values were similar to values reported in other papers [34,35]. Considering the utility value from a published study in India [35], the scenario analysis was conducting by varying the EQ-5D utility score to 0.925 for mild and moderate cases. Additional QALYs gained among incidence cases for N95 masks in comparison to surgical masks were 71 with 3 deaths averted.

## Discussion

There is a lack of published literature on the cost-effectiveness of preventive measures of N95 and surgical masks for COVID-19 as compared to no intervention among HCWs, the authors believe this economic evaluation is the first to assess this in the Indian context among the population of HCWs. Both of the considered interventions were dominant compared to no mask based on the model estimates, at both of the analyzed CETs. N95 masks were also dominant compared to surgical masks. A study conducted in China estimated that the incremental cost ($490–$1,230) to avoid a chronic respiratory illness case with the use of N95 compared to medical masks [36]. N95 masks also appeared to result in fewer deaths due to COVID-19, compared to surgical masks and no mask in our model. Similar to N95, the use of a surgical mask was also shown to be cost-effective in comparison to no mask. Literature has shown the effectiveness of masks to prevent the transmission of respiratory infections among HCWs wearing a surgical mask with hand hygiene [11].

The strength of our study was that primary data were collected for estimating the QALYs and compliance rate of mask-wearing among HCWs. A previously published model was used to ensure the robustness of the methods [15]. The full model validation was done in the original model article [10], where the model was fitted to cumulative mortality data compiled by the Center for Systems Science and Engineering at Johns Hopkins University (2020). Parameter fitting was performed using a nonlinear least squares algorithm. In our study, we calibrated the transmission, recovery and death rates parameters for modelling COVID-19 among the healthcare professionals. The model obtained the estimated incidence of COVID-19 of 3–8%, falling within the range of incidence reported among the healthcare staff in India setting [37]. Another strength of the study is that on considering the lower willingness-to-pay threshold for India ($115-$770), even at the lower estimated threshold of India’s GDP per capita, the conclusions of the results remained unchanged.

An uncertainty analysis was also conducted which showed that the model results were not particularly sensitive to the uncertainty surrounding the efficiency parameters of masks and ICER values. This shows that the model results were robust, even to variation of parameters of intervention efficiency. However, considering the complex nature of the model, sensitivity analysis for all parameters was not conducted.

The simplification and uncertainties in parameter estimation are one of the limitations of the study. β was assumed to be fixed in the study. The study considers COVID-19 patients to be an exogenous risk to HCWs, and is specific to the emergency phase of the pandemic when transmission was highest. The threat appears to be constant over time rather than fluctuating in a tertiary care institute designated as ’COVID-19 hospital’. However, uncertainty analysis was conducted considering the lower and higher range of the β. Another limitation was that a proxy method for calculating incremental QALYs based on life expectancy and mortality was used. However, given the available data, this appeared to be the most accurate and reliable way of estimating incremental QALYs. The εo appears more frequently than εi in the model, assuming outward effectiveness to be of greater importance in the hospital scenario. The notion behind the assumption is prevention of the asymptomatically HCWs from transmitting disease considering outward effectiveness of the masks. The model predicted less mortality numbers in the intervention arms, as the mask compliance data was collected during the middle of COVID-19 among HCWs and likely represents best-case scenarios in terms of mask compliance (90%). But it is worthwhile to mention that the compliance may not remain the same as was during the pandemic. Hence, no mask scenario was also kept in the model.

The scope of the current study is limited to the cost-effectiveness of masks (N95/surgical) considering the same as a source control and primary prevention of COVID-19 among HCWs in a tertiary care institute of national importance in India. The study didn’t consider other non-pharmacological interventions (NPIs) such as hand hygiene and social distancing, as these interventions were already in place in all scenarios.

This study evaluated the impact of masking among HCWs but did not explore the consequences of HCWs wearing masks on the wider general population. However, this would have likely made mask-wearing even more cost-effective than was estimated in the model, as the masks would reduce COVID-19 incidence even further. But these findings contrast to an Indian study that examined the cost-effectiveness of mask-wearing with hand hygiene for the general population in India and found that they were not cost-effective due to higher costs and relatively lower effectiveness in preventing the COVID-19 [11]. This study showed that considering fixed transmission rate, adoption of mask efficiency as 20%, 50% and 80% reduces the cumulative relative mortality to 41%, 79% and 94% respectively. Eikenberry et al [10] showed that in Washington state, considering a fixed transmission rate, adoption of 20%, 50%, and 80% effective masks reduces cumulate relative mortality to 65%, 91%, and 95% respectively.

In India, the guidelines on Clinical Management of COVID-19 came into effect on 31^st^ March 2020 and recommended specific infection prevention and control practices for healthcare workers [38]. According to these guidelines, surgical masks should be used when working within 1–2 meters of the patient and HCWs performing aerosol-generating procedures should be provided with a PPE kit and fit-tested particulate respirators (N95). The price of masks (surgical masks & N95) was capped by Essential Commodities Act, 1955 in March 2020 [39]. All state governments of India were ordered to ensure sufficient availability of the masks and prices not to exceed the maximum retail price. The Union ministry of Health and Family Welfare issued a circular in April 2020 that productions, procurement of masks, PPE, ventilators etc. will be done by the empowered group of central government.

Presently, most of the COVID-19 related restrictions have been lifted by the authorities in the States and union territories of India based on the advice of the Centre to consider discontinuing the COVID-19 containment measures in view of a sharp decline in the number of fresh cases of the infection in the country [40]. Our study may be useful to provide evidence for future research and policymaking for other pandemic respiratory diseases similar to COVID-19 [41] during an emergency phase, similar to how data from previous studies on severe acute respiratory syndrome (SARS) was used in the simulation of COVID-19.

Most of the literature on the cost-effectiveness of masks for prevention of COVID-19 among HCWs comes from high-income countries. This research adds to a growing body of health economic literature in the Indian setting and can inform future studies of a similar nature in this setting. This analysis considered a population of HCWs and associated costs of masks using data from an actual facility in a tertiary care setting in India. The findings illustrate that provision of N95 for HCWs may be beneficial in reducing harm to HCWs during a crisis like COVID-19, and was more cost-effective than surgical masks. Based on the current study, the Ministry of Health and Family welfare department in India should mandate to provide N95 masks for all HCWs, as this was the cheapest and most effective strategy. Adopting this approach would save lives of HCWs and money in a resource constrained setting of India.

However, it is imperative that in addition to the provision of N95 masks, consideration should be given to the attitude of the HCWs towards mask use, compliance, comfort correct fitting, long hours of use, potential risks during doffing, weather conditions, disposal, adverse effects or any associated dermatological or oral concerns and accessibility of the masks to all HCWs (health professionals, health associate professionals, personal care workers in health services, health management and support personnel, and other health service providers) due to under-funded healthcare infrastructure. Further studies may be conducted on the impact of mask-wearing in other high-risk populations in India e.g., older people and individuals with chronic medical conditions like diabetes and hypertension as these masks may be cost-effective interventions in these populations too.

## Supporting information

S1 Fig Differential equations for a model with no mask and with mask [15]. (DOCX)

S1 Table QALYS of incident cases in different health states. (DOCX)
