# Supplementary material for: Cost-effectiveness analysis of surgical masks, N95 masks compared to wearing no mask for the prevention of COVID-19 among health care workers: Evidence from the public health care setting in India
Source: PLoS One. 2024 May 20;19(5):e0299309. doi: 10.1371/journal.pone.0299309 (PMC11104672; doi:10.1371/journal.pone.0299309)

## **S1 Fig: Differential equations for a model with no mask and with mask**^[15]^

Susceptible S(t), exposed E(t), symptomatic infectious I(t), hospitalized H(t), asymptomatic infectious A(t), recovered R(t), and cumulative deaths D(t), Transmission rate (β), Recovery rate (λ), Transition exposed to infectious (σ), infectiousness factor for asymptomatic carriers (η), fraction of infections that become symptomatic (α), Rate of hospitalisation (ϕ), Recovery rate per day, asymptomatic (γa), recovery rate per day, symptomatic (γI), Recovery rate, hospitalised (γH), Death rate (δ)

Symptomatic infectious (I_M_+I_U_), Asymptomatic infectious (A_U_+A_M_), Hospitalised (H_U_+H_M_), Recovered (R_U_+R_M_), and Death (D_U_+D_M)._ The total number of COVID-19 cases in the respective compartments are calculated as the sum of Symptomatic infectious (I_M_+I_U_), Asymptomatic infectious (A_U_+A_M_), Hospitalised (H_U_+H_M_), Recovered (R_U_+R_M_) and Death (D_U_+D_M_) over a one-year time horizon, and the cases presented for each of the intervention is obtained as the difference in the total cases between the intervention and no mask. The total number of mild COVID-19 HCWs is calculated as the sum of symptomatic infectious (I_M_+I_U_) and the total number of asymptomatic COVID-19 HCWs is the sum of asymptomatic infectious (A_U_+A_M_). HCWs who don’t wear masks subscripted with U and M respectively.

### No mask


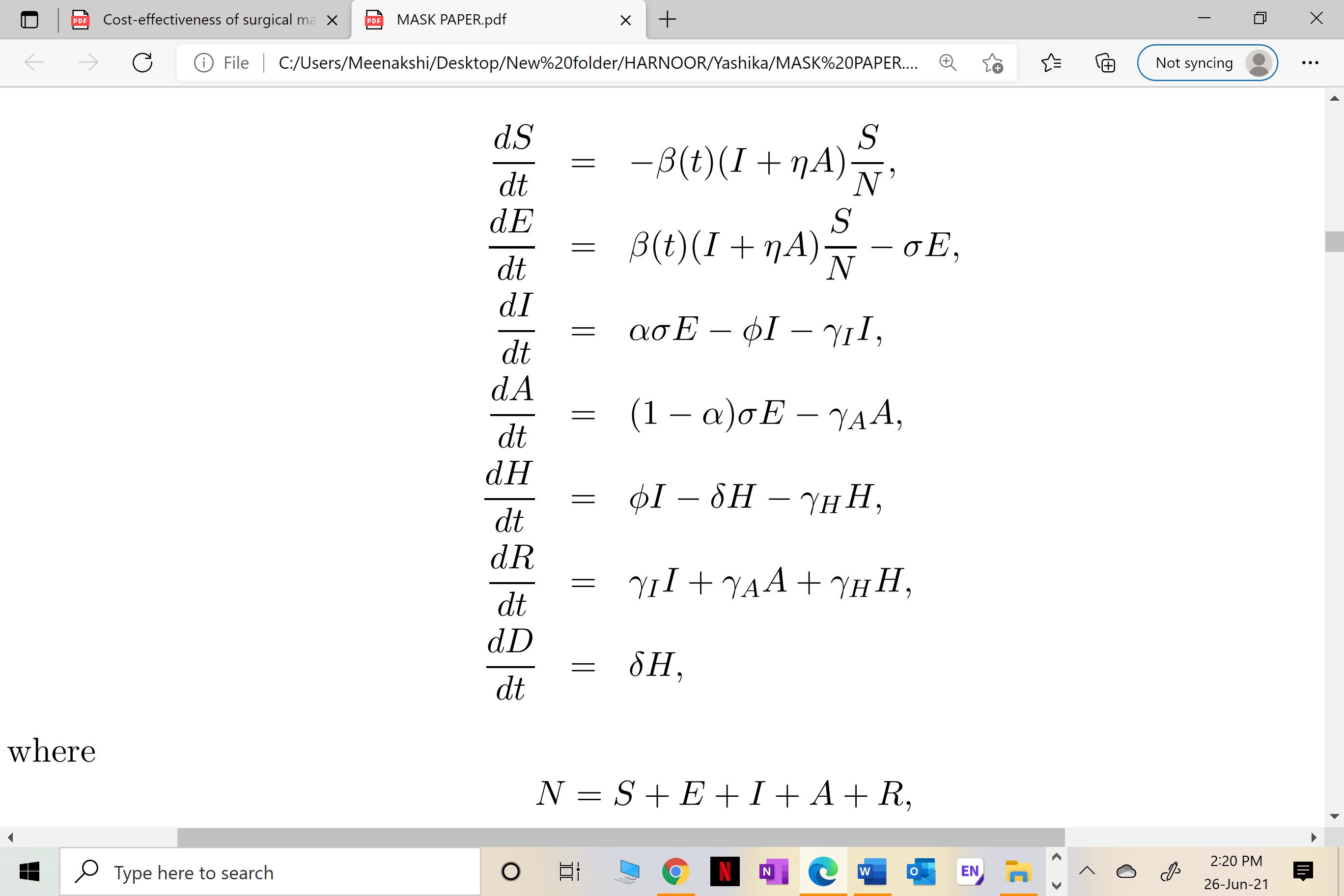


## With mask


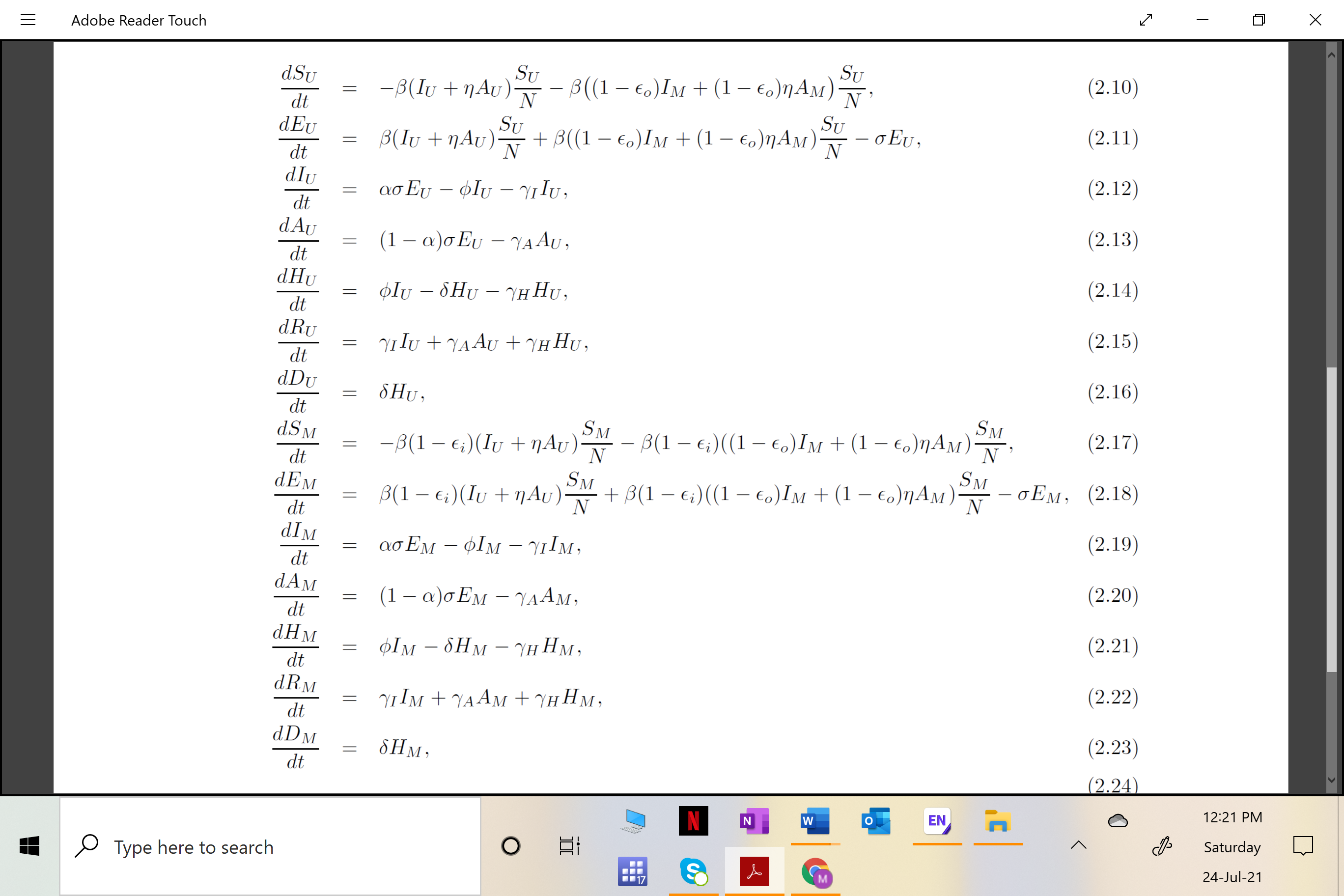

Supplement: S1 Fig — (DOCX) [file pone.0299309.s001.docx]
