# Supplementary material for: Cost-effectiveness analysis of surgical masks, N95 masks compared to wearing no mask for the prevention of COVID-19 among health care workers: Evidence from the public health care setting in India
Source: PLoS One. 2024 May 20;19(5):e0299309. doi: 10.1371/journal.pone.0299309 (PMC11104672; doi:10.1371/journal.pone.0299309)
Supplement: S1 Table — (DOCX) [file pone.0299309.s002.docx]

## **S2 Table: QALYS of incident cases in different health states**

|  | **Symptomatic** | **Hospitalization** | | | **QALYS gained from deaths averted** | **Total QALYS** |
| --- | --- | --- | --- | --- | --- | --- |
|  |  | **Moderate** | **Severe** | **Critical** |  |  |
| No mask-N95 | 15.78 | 2.12 | 1.96 | 1.21 | 868.98 | 890.04 |
| No mask-surgical | 14.50 | 2.04 | 1.88 | 1.16 | 797.59 | 817.17 |
| Surgical-N95 | 1.28 | 0.08 | 0.07 | 0.05 | 71.38 | 72.87 |
